# Percutaneous epididymal sperm aspiration as a method for sperm retrieval in men with obstructive azoospermia seeking fertility: operative and laboratory aspects

**DOI:** 10.1590/S1677-5538.IBJU.2015.0064

**Published:** 2015

**Authors:** Sandro C. Esteves

**Affiliations:** 1 ANDROFERT - Andrology and Human Reproduction Clinic, Campinas, SP, Brasil

## Abstract

**Introduction:**

Congenital bilateral absence of vas deferens (CBAVD) is a non-treatable cause of obstructive azoospermia (OA). However, the affected men can father children by undergoing sperm retrieval (SR) and intracytoplasmic sperm injection (ICSI).

**Materials and Methods:**

This video describes percutaneous epididymal sperm aspiration (PESA), performed on a 36 year-old male with CBAVD. In PESA the goal is to obtain epididymal fluid. A hypodermic needle attached to a 1 cc syringe is inserted through the skin into the corpus or caput epididymis. Gentle negative pressure is applied to aspirate the epididymal fluid, which is sent to the laboratory for examination.

**Results:**

Total number of spermatozoa retrieved after a single puncture was 3.5 million sperm, of which 29% were motile. Motile spermatozoa with normal morphology were selected and injected into the oocyte cytoplasm, while excess retrieved sperm were cryopreserved. The operative time was 10 minutes. The patient recovered his normal activities within the next day and no complications were recorded. In a series involving 32 men with CBAVD, success rate at obtaining motile sperm by PESA was 96.8%, with a complication rate of 3.1%. ICSI carried out with spermatozoa retrieved by PESA resulted in a live birth rate of 34.4% per attempt. The short-term outcome of resulting offspring was comparable with those obtained in other categories of OA.

**Conclusion:**

PESA is a simple, quick, and successful procedure to retrieve sperm from men with OA due to CBAVD. Retrieved sperm can be successfully used to generate healthy offspring with the aid of ICSI.

## ARTICLE INFO

**ARTICLE INFO Available at:** **www.brazjurol.com.br/videos/july_august_2015/Esteves_817_818video.htm**

**Int Braz J Urol. 2015; 41 (Video #6): 817-8**
