# Supplementary material for: Percutaneous epididymal sperm aspiration as a method for sperm retrieval in men with obstructive azoospermia seeking fertility: operative and laboratory aspects
Source: Int Braz J Urol. 2015 Jul-Aug;41(4):817–8. doi: 10.1590/S1677-5538.IBJU.2015.0064 (PMC4757014; doi:10.1590/S1677-5538.IBJU.2015.0064)
Supplement: Related texts

# Letter: EDITORIAL COMMENT

**Authors:** Philippe E. Spiess

**Affiliations:** Video Section Editor, International Brazilian Journal of Urology. E-mail: Philippe.Spiess@moffitt.org

In this very nice video, the authors highlight the clinical merit of percutaneous epididymal sperm aspiration (PESA) in the management of obstructive azoospermia. The video highlights not only the key features of this technique but depict within a consecutive cohort of 32 male patients that excellent outcomes can be achieved, with a very acceptable low complication rate. It clearly validates PESA as a reliable and excellent choice in the management of obstructive azoospermia. There are clearly many factors which must be considered in deciding which therapeutic intervention is selected in the management of obstructive azoospermia such as the success in achieving high concentrations of motile sperm while imparting minimal to no risk. Clearly, PESA achieves these endpoints quite successfully and hence constitutes an excellent choice in fertility management provided intracytoplasmic sperm injection is readily available to couples thereafter and hopefully resulting in a successful conception.

*Philippe E. Spiess, M.D.* *Video Section Editor,* *International Brazilian Journal of Urology* *E-mail: Philippe.Spiess@moffitt.org*

# Letter: EDITORIAL COMMENT

**Authors:** Tariq Hakki

**Affiliations:** Advanced Urology, Male Reproductive Medicine and Surgery, 1557 Janmar Road, Snellville, GA 30097. E-mail: tariq7@gmail.com

Sperm can be obtained by microepididymal sperm aspiration (MESA), percutaneous sperm aspiration (PESA), and testicular sperm extraction (TESE), from patients with congenital absence of the vas deferens (CBAVD). The authors of the video on PESA as a method for sperm retrieval in obstructive azoospermia nicely demonstrate this technique in men with CBAVD. These patients have an abnormal epididymis from this condition making the retrieval more challenging (1). In their case series of 32 men with CBAVD, success rate at obtaining motile sperm by PESA was 96.8%, with a live birth rate of 34.4% per attempt. The short-term outcome of resulting offspring was comparable with those obtained in other types of obstructive azoospermia. Percutaneous aspiration of sperm is an effective, safe, and reproducible means of obtaining adequate sperm in men who have obstructive azoospermia due to CBAVD.

*Tariq Hakki, MD* *Advanced Urology* *Male Reproductive Medicine and Surgery* *1557 Janmar Road* *Snellville, GA 30097* *E-mail: tariq7@gmail.com*

## References

[1] Dohle GR, Ramos L, Pieters MH, Braat DD, Weber RF (1998). Surgical sperm retrieval and intracytoplasmic sperm injection as treatment of obstructive azoospermia. Hum Reprod.
